# Impact of intestinal bacteria on biofilm formation, motility, virulence, and gene expression in *Vibrio parahaemolyticus*

**DOI:** 10.1128/spectrum.00789-25

**Published:** 2025-07-07

**Authors:** Qinglian Huang, Xin Shen, Xi Luo, Miaomiao Zhang, Xue Li, Shenjie Ji, Yiquan Zhang, Renfei Lu

**Affiliations:** 1 Department of Clinical Laboratory, Qidong People's Hospital 580535 , Qidong, Jiangsu, China; 2 Department of Clinical Laboratory, Nantong Third People’s Hospital, Affiliated Nantong Hospital 3 of Nantong University 611612, Nantong, Jiangsu, China; 3 School of Medicine, Nantong University 117814, Nantong, Jiangsu, China; Griffith University - Gold Coast Campus, Gold Coast, Queensland, Australia

**Keywords:** *Vibrio parahaemolyticus*, *Escherichia coli*, biofilm formation, swimming, RNA-seq, gene expression

## Abstract

*Vibrio parahaemolyticus*, a foodborne pathogen, is a leading cause of acute gastroenteritis in humans. In contrast, non-pathogenic *Escherichia coli* is a common commensal bacterium residing in the human gut. Despite their co-occurrence during infection, the interaction between these two species remains poorly understood. This study revealed that *V. parahaemolyticus* outcompetes *E. coli* under co-culture conditions, demonstrating superior growth efficiency and competitive dominance. Co-cultivation stimulated biofilm formation in both species, with *E. coli* culture supernatant specifically enhancing biofilm development in *V. parahaemolyticus*. Nevertheless, *V. parahaemolyticus* dominated mixed microbial colonies. Additionally, co-cultivation reduced *V. parahaemolyticus* cell adhesion and swimming motility but heightened its cytotoxicity. RNA-seq analysis identified 629 differentially expressed genes in co-cultured *V. parahaemolyticus* compared to monocultured cells, including 290 upregulated and 339 downregulated genes. Genes related to flagella and type III secretion system 1 were downregulated, while those for exopolysaccharides, type IV pili, and type VI secretion system 2 were upregulated. Notably, eight genes involved in c-di-GMP metabolism showed significant changes, with seven being upregulated. Additionally, 22 putative transcriptional regulators exhibited differential expression, indicating tightly coordinated gene expression during interspecies interactions. These findings illuminate the dynamic interplay between *V. parahaemolyticus* and gut commensals, providing insights into pathogen behavior in polymicrobial environments. This work lays the groundwork for future studies on gut microbiota-pathogen interactions and their implications for infectious disease mechanisms.

**IMPORTANCE:**

This study reveals critical insights into the interplay between the gut commensal bacterium *E. coli* and the foodborne pathogen *V. parahaemolyticus*, offering novel perspectives on how gut microbiota influence pathogen behavior. *V. parahaemolyticus* outcompetes *E. coli* in co-culture, highlighting its adaptability and potential to disrupt gut microbial balance during infection. The *E. coli* culture supernatant enhances *V. parahaemolyticus* biofilm formation. Co-cultivation reduces *V. parahaemolyticus* motility but increases cytotoxicity. RNA-seq analysis identified 629 differentially expressed genes, including those associated with downregulated flagellar/T3SS1 systems and upregulated exopolysaccharide/T6SS2 pathways. These findings advance our understanding of gut microbiota-pathogen dynamics and could inform strategies to mitigate *V. parahaemolyticus* infections by targeting interspecies signaling or virulence pathways.

## INTRODUCTION

The gut microbiota is a community of microorganisms, including fungi, viruses, bacteria, and protozoa, that reside in the human gastrointestinal tract (1). These microorganisms interact intricately with the host and significantly impact health. They enhance the intestinal mucosal barrier to defend against pathogens; metabolize essential nutrients such as carbohydrates, fats, proteins, and vitamins; regulate immune function; and contribute to resistance against tumor development (2, 3). However, disruptions to gut microbiota balance can allow pathogenic bacteria to exploit this vulnerability, leading to disease and threatening host health (3). Non-pathogenic *Escherichia coli* is a common commensal bacterium in the human gut (4). This Gram-negative, facultative anaerobe colonizes the intestinal tract shortly after birth and persists throughout the host’s lifespan (4). *E. coli* consumes oxygen during growth, creating an anaerobic microenvironment that favors bacteria like *Bifidobacterium*, which are critical for maintaining gut microbiota homeostasis (4). Under normal conditions, *E. coli* plays a key role in the intestinal ecosystem by inhibiting competing microorganisms and protecting against pathogenic invaders (4).

*Vibrio parahaemolyticus* is the most common foodborne pathogen in coastal areas, widely distributed in nearshore seawater, seabed sediments, and seafood such as crab and shellfish (5). It primarily causes acute gastroenteritis, characterized by clinical symptoms including vomiting, abdominal pain, and diarrhea (5). The major virulence factors of *V. parahaemolyticus* include thermostable direct hemolysin (TDH), type III secretion systems (T3SS1/T3SS2), type VI secretion systems (T6SS1/T6SS2), adhesion factors, lipopolysaccharides, proteases, and iron acquisition systems (6, 7). These factors exhibit distinct physiological functions and play diverse roles during the infection process and pathogenesis of the bacterium (6, 7). *V. parahaemolyticus* also forms biofilms, multicellular structures that adhere to surfaces (8). Biofilms consist of bacterial cells embedded in an extracellular matrix composed of exopolysaccharides (EPS), extracellular proteins, lipopolysaccharides, and extracellular DNA (9). Biofilm formation is a key survival strategy for *V. parahaemolyticus* under adverse conditions and contributes to the challenge of eliminating it from seafood products (8). Genetic loci critical for biofilm formation in *V. parahaemolyticus* include those involved in EPS production, flagellar assembly, and type IV pili synthesis (9).

*V. parahaemolyticus* cannot grow in environments with a pH lower than 4.5 (10), whereas human gastric acid typically ranges from pH 1 to 3. Biofilm formation and co-ingestion with food particles are key factors enabling this bacterium to resist gastric acid damage and reach the intestinal tract. Upon entry, *V. parahaemolyticus* interacts with the gut microbiota, altering the microbiota’s composition and abundance (11, 12). This disruption of gut microbial homeostasis allows *V. parahaemolyticus* to proliferate and dominate the ecosystem (11, 12). Furthermore, the pathogen releases virulence factors that trigger cytotoxic and entero-toxic responses, ultimately compromising the intestinal mucosal barrier. Conversely, the gut microbiota also plays a critical role in countering *V. parahaemolyticus* invasion. For instance, non-pathogenic *E. coli* BL21 expressing MAM7 effectively inhibits *V. parahaemolyticus* infectivity, and pre-incubation with BL21-MAM7 significantly reduces the pathogen’s cytotoxicity (13). However, how the gut microbiota influences *V. parahaemolyticus* gene expression and biological behaviors remains poorly understood. In this study, we investigated the interactions between *E. coli* and *V. parahaemolyticus*. Our findings indicate that co-cultivation with *E. coli* significantly influences *V. parahaemolyticus* biofilm formation, motility, virulence, and gene expression.

## MATERIALS AND METHODS

### Bacterial strains and growth conditions

The strains used in this study were *V. parahaemolyticus* RIMD2210633 (14) and *E. coli* ATCC25922. Both strains were grown in Luria-Bertani (LB) broth, which is composed of 1% tryptone (OXOID, UK), 0.5% yeast extract (OXOID, UK), and 1% NaCl (Merck, Germany), at 37°C with shaking in glass test tubes. An overnight culture was diluted 50-fold into fresh LB broth and incubated at 37°C with shaking until reaching an optical density at 600 nm (OD_600_) of 1.0 (~5 × 10^8^ CFU/mL), which was termed the bacterial seed. To distinguish *V. parahaemolyticus* from *E. coli* in co-culture, 100 µg/mL ampicillin was used to selectively isolate *V. parahaemolyticus*, as the former is resistant to 100 µg/mL ampicillin, whereas the latter is sensitive to it (15).

### Measurement of growth curves

The bacterial seed of *V. parahaemolyticus* (or *E. coli*) was diluted 1:1,000 (or 1:10) into 10 mL of fresh LB broth containing 0, 5, 10, or 20 µL culture supernatant (CS) from *E. coli* (or *V. parahaemolyticus*) grown overnight. After mixing, aliquots were distributed into a 96-well plate (200 µL per well), with five replicates per condition. For the co-culture growth curves of *V. parahaemolyticus* and *E. coli*, the bacterial seed of *V. parahaemolyticus* was diluted 1:1,000 and that of *E. coli* was diluted 1:10 into 10 mL LB broth in a single test tube. The final ratio of *E. coli* to *V. parahaemolyticus* in the mixture was approximately 100:1, simulating an intestinal environment during infection (16). The OD_600_ values of each well were monitored at 30 min intervals using a microbial growth curve analyzer (MGC-200, Ningbo Scientz Biotechnology Co., Ltd., Ningbo, China) to create growth curves. Growth conditions were maintained at 37°C with shaking at 800 rpm (17).

### Colony counting

*V. parahaemolyticus* exhibits high resistance to ampicillin, whereas *E. coli* demonstrates high sensitivity to this antibiotic (18–20). Based on this distinction, the populations of *V. parahaemolyticus* and *E. coli* in co-culture were quantified. The methodology is as follows: a 100 µL aliquot of the bacterial co-culture was serially diluted (10-fold), spread onto LB plates, and incubated at 37°C for colony counting to determine the total CFUs of both species. To selectively quantify *V. parahaemolyticus*, an aliquot was also spread onto LB plates supplemented with 100 µg/mL ampicillin. By subtracting the CFU count of *V. parahaemolyticus* from the total CFU count, the CFU count of *E. coli* in the co-culture was calculated.

### Quantitative assessment of biofilm formation using crystal violet staining

For single-species biofilms, the bacterial seed of *V. parahaemolyticus* or *E. coli* was diluted 1:1,000 or 1:10, respectively, in 10 mL of fresh LB broth. Aliquots (1 mL per well) were transferred to a 24-well plate, with eight replicate wells per strain. For co-culture biofilms, *E. coli* and *V. parahaemolyticus* were co-inoculated into 10 mL LB broth at a 100:1 ratio (*E. coli:V. parahaemolyticus*), mixed thoroughly, and aliquoted (1 mL per well) into a 24-well plate. To assess biofilm interactions, 1 mL of *V. parahaemolyticus* or *E. coli* suspension was added to individual wells of a 24-well plate and incubated at 37°C with shaking for 12 h. After removing the culture, wells were gently washed with 1 mL sterile phosphate-buffered saline (PBS). Fresh suspensions of the opposing strain (1 mL *E. coli* or *V. parahaemolyticus*, respectively) were added, followed by a 12 h incubation at 37°C. To assess the effect of CS on biofilm formation, the CS of *E. coli* or *V. parahaemolyticus* cultured in LB broth at 37°C with shaking for 12 h was collected, filtered to remove bacterial cells, and stored for further use. Heat-treated CS (100°C for 10 min) was prepared for comparative analysis. For *V. parahaemolyticus* biofilm assays, fresh or heat-treated *E. coli* CS was added to 1 mL of *V. parahaemolyticus* suspension in a 24-well plate at final concentrations of 5%, 10%, and 20%. For *E. coli* biofilm assays, *V. parahaemolyticus* CS was added to 1 mL of *E. coli* suspension in a 24-well plate at final concentrations of 10%, 20%, 40%, and 60%. The control group received an equal volume of sterile LB broth. Mixtures were incubated at 37°C with shaking for 12 h. For biofilm quantification (21), biofilms were washed three times with distilled water to remove unattached cells and then stained with 0.1% CV solution for 20 min. Bound CV was solubilized in 20% acetic acid, and absorbance was measured at 570 nm (OD_570_). Cell density in the planktonic phase was determined via OD_600_ measurements. Relative biofilm formation was expressed as the ratio of OD_570_ to OD_600_.

### Colony morphology

A volume of 2 µL of bacterial seed stock of *V. parahaemolyticus* or *E. coli*, or a mixture of the bacterial seed stocks of *V. parahaemolyticus* and *E. coli* at 1:100 CFU ratio, was spotted onto LB plates and incubated at 37°C for 24 h. A photograph of individual colonies was taken.

### Detection of *V. parahaemolyticus* via polymerase chain reaction assay

A small amount of a mixed colony (consisting of *E. coli* and *V. parahaemolyticus*) from the colony morphology assay was randomly collected and suspended in aseptic PBS buffer. The mixture was thoroughly homogenized and subjected a 10-fold dilution. Subsequently, the diluted sample was spread onto LB plates and incubated at 37°C for 24 h. Single colonies were randomly selected, resuspended in sterile deionized water, and boiled at 100°C for 5 min. After centrifugation, the supernatant was applied as a template for PCR. The species-specific marker gene *toxR* was selected as the target gene to verify *V. parahaemolyticus* colonies (18). The primer sequences for the *toxR* gene were GTCTTCTGACGCAATCGTTG and ATACGAGTGGTTGCTGTCATG (18). The PCR mixture consisted of 10 µL of 2 × *Taq* PCR Mastermix (Tiangen, China), 2 µL of template DNA, 1 µL of primer pair (10 µM each), and 7 µL of water. PCR amplification was conducted under the following conditions: pre-denaturation at 95°C for 5 min, followed by 35 cycles of denaturation at 94°C for 50 s, annealing at 54°C for 50 s, and extension at 72°C for 50 s, with a final extension at 72°C for 5 min. PCR amplicons were analyzed by electrophoresis on a 1% agarose gel.

### Motility assays

For the swimming assay (22), 2 µL of bacterial seed stock of *V. parahaemolyticus* or *E. coli*, or a mixture of the bacterial seed stocks of *V. parahaemolyticus* and *E. coli* at a 1:100 CFU ratio, was inoculated into semi-solid LB plates that contained 0.2% (wt/vol) Difco Noble agar (BD Biosciences, USA). For the swarming assay (22), 2 µL of these bacterial seed stocks was spotted onto solid LB plates that contained 1.8% (wt/vol) Difco Noble agar. The diameters of the motility zones were measured after incubation at 37°C for 7 h and 48 h, respectively.

### Cell adhesion assay

Cell adhesion assays were performed as previously described (23). Briefly, HeLa cells were cultured in Dulbecco’s modified Eagle medium (DMEM; Gibco, USA), enriched with 10% fetal bovine serum (FBS) (Invitrogen, USA), and maintained at 37°C with 5% CO_2_. Bacterial seed stocks of *V. parahaemolyticus* and *E. coli* were collected by centrifugation, washed twice with DMEM, and resuspended in DMEM. For *V. parahaemolyticus* adhesion, HeLa cells were infected at a multiplicity of infection (MOI) of 10. For *E. coli* adhesion, HeLa cells were infected at an MOI of 1,000. For co-infection with mixed bacteria, *V. parahaemolyticus* and *E. coli* were mixed at a 1:100 CFU ratio. HeLa cells were then infected at an MOI of 10 relative to the *V. parahaemolyticus* count. Bacteria cells were also added to empty wells to ascertain the initial bacterial count. After a 60 min incubation at 37°C in 5% CO_2_, monolayers were washed three times with PBS to eliminate unbound bacteria. Cells were lysed with 0.5% Triton X-100. Lysates and initial bacterial samples were serially diluted 10-fold and plated on LB agar for colony counting. Percent adherence was calculated as (adherent bacterial cells/input bacterial cells) 100×.

### Cytotoxicity assay

Cytotoxicity against HeLa cells was conducted as previously described (23). Briefly, HeLa cells were infected with 10^6^ CFU of *V. parahaemolyticus*, 10^8^ CFU of *E. coli*, or a combination of 10^6^ CFU *V*. *parahaemolyticus* and 10^8^ CFU *E. coli*, for 3 h at an MOI of 2.5 (based on *V. parahaemolyticus* count). Lactate dehydrogenase (LDH) release was measured using the CytoTox 96 Non-radioactive Cytotoxicity Assay kit (Promega, USA) according to the manufacturer’s instructions.

### RNA sequencing

The bacterial seed stock of *V. parahaemolyticus* was transferred to 5 mL of fresh LB broth diluted 1:1,000. Alternatively, *V. parahaemolyticus* seed stock (diluted 1:1,000) and *E. coli* seed stock (diluted 1:10) were inoculated into 5 mL of fresh LB broth. Three replicates were set for each condition. Cultures were incubated at 37°C in a 24-well plate with shaking for 6 h. Bacterial cells were harvested, and total RNA was extracted using TRIzol Reagent (Invitrogen, USA). Ribosomal RNA (rRNA) depletion and mRNA enrichment were performed using the Illumina/Ribo-Zero rRNA Removal Kit (bacteria). cDNA libraries were constructed and sequenced on the Illumina HiSeq platform at GENEWIZ Biotechnology Co. Ltd. (Suzhou, China). Bioinformatic analysis was performed as previously described (22). *V. parahaemolyticus* cultured alone served as the reference group, and *V. parahaemolyticus* co-cultured with *E. coli* was the experimental group. Genes with |log_2_(fold change)| ≥ 1 and *P* < 0.05 were considered significantly differentially expressed.

### Quantitative real-time PCR

Following the RNA-seq culture conditions, bacteria were cultured, and total RNA was extracted. cDNA was generated from 1 µg of total RNA using the FastKing gDNA Dispelling RT SuperMix (Tiangen Biotech, China). qPCR was performed on a LightCycler 480 system (Roche, Switzerland) with SYBR Green master mix. Relative gene expression was calculated using the 2^−ΔΔCt^ method (24), normalized to the 16S rRNA gene. Primers are listed in Table 1.

**TABLE 1:** Primers used in this study

Target	Primers (forward/reverse, 5'−3')
VP0788	GGCTGAAGGTGCGATGAAC/AGACGACGACCACCGAATG
VP2258	TGCAGGTCTACAGATTTCAAACC/CCTTCAGCAACCTGTGCAA
VP2259	ACAACGTATGCGTGACCTGT/TCAGCACCGATTTGGAACGA
*cpsA*	GAGAGCGGCAACCTATATCG/CGCCACGCCAACAGTAATG
*cpsE*	GTCTCTTGGCGTGCTTATC/GAGCCGACTTTACCCATTTG
*hcp2*	TAAAGGTGAAGCGACAGCG/AATCATATAGGCGTGTTGC
VPA1043	TGACCATAACGAGTTTCCAC/TTTAATCAATTCGCCGTGAG
VPA1044	ATAGCAGCGATAGCGGAG/TTTGAGACAGTTTTGTATCC
*gepA*	GACCACCTCAATAGTTATCTG/TAAGTAGGCTTGGACATCTC
VP1979	TTAAAGCCAGCGATGTAAACCC/GGCGAATCTGTTCTAACGCAAA
VPA0360	TCGTTCTTTACCTACGCCTTA/TGCCAATAACACTCGATAGAGC
VPA0518	GAAACATTAACGCAGCAAGCC/TCACCAATGTAGTTCCCGTTG
VPA0594	GGGTTAGTATCGTTGCTGACTG/ATGCCGAGCGACACATTATTC
VPA0878	TAAAGATGCGCTCACAACAAA/CATCGAGCTTACTTCTTTCAT
VP1649	TGAGAAAGAACACACCAAG/TCCGAGATTTTGCTACCCG
VP2710	GCATTCCTGTAATCTCAACC/AGTTCTTCCAAACCAATGGC
*qsvR*	TACACCGCCACCCATAACG/AGCCATTCTCGCCAGGTATG
*cpsQ*	GCCTGAAATCCTAATGCTC/AGTGTCAGAAGGTGTATCAAC
*cpsS*	GAGGGCAGTCTACAGTCATC/ACGAGCAAACACTTCATCTG
16S rRNA	GACACGGTCCAGACTCCTAC/GGTGCTTCTTCTGTCGCTAAC

### Statistical methods

All experiments (excluding RNA-seq) were repeated at least three times with triplicate samples. Data are presented as mean ± standard deviation (SD). One-way ANOVA was performed to assess statistical significance, with a *P*-value of less than 0.05 considered significant.

## RESULTS

### Co-cultivation of *V. parahaemolyticus* and *E. coli*

**Fig 1:**
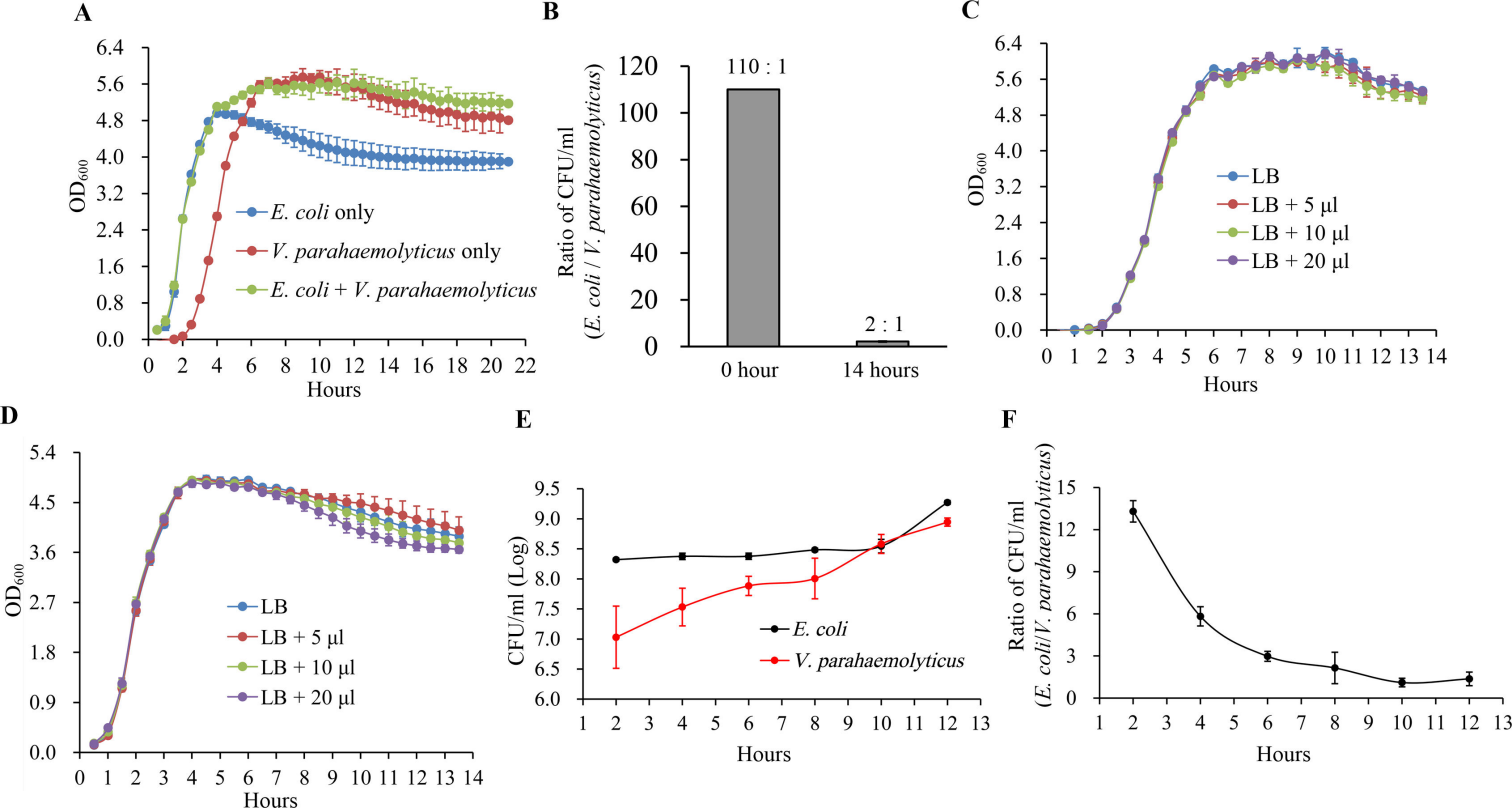
Growth of *V. parahaemolyticus* and *E. coli* under monoculture and co-culture conditions. (**A**) Growth curves. The growth curves of *V. parahaemolyticus* and *E. coli* were evaluated using a microbial growth curve analyzer (MGC-200) in a 96-well culture plate. The OD_600_ of each bacterial culture was measured every 30 min to track growth. The cultures were incubated under standardized conditions at a temperature of 37°C with an orbital shaking frequency of 800 rpm. (**B**) The ratio of CFU of *E. coli* to *V. parahaemolyticus* in co-cultures under different incubation durations. (**C**) The effect of *E. coli* CS on the growth of *V. parahaemolyticus*. (**D**) The effect of *V. parahaemolyticus* CS on the growth of *E. coli*. (**E**) The variations in the CFU of *E. coli* and *V. parahaemolyticus* in co-cultures. *V. parahaemolyticus* and *E. coli* were inoculated into LB broth at a ratio of 1:100 and then incubated statically at 37°C. The CFU of both *E. coli* and *V. parahaemolyticus* in the culture was measured every 2 hours. Additionally, the ratio of CFUs between *E. coli* and *V. parahaemolyticus* was also calculated (**F**).

In order to assess the impact of *E. coli* on the growth of *V. parahaemolyticus*, we first determined the growth curves for cultures grown separately and in co-culture at a 100:1 ratio of *E. coli* to *V. parahaemolyticus*. As shown in Fig. 1A, during the logarithmic phase, the OD_600_ values of *V. parahaemolyticus* cultured alone lag behind those of *E. coli* cultured alone and the co-culture. The primary cause of this phenomenon may be the different initial quantities of *E. coli* and *V. parahaemolyticus*. The CFU of *E. coli* was ~100 times that of *V. parahaemolyticus*, and theoretically, *V. parahaemolyticus* has a longer lag phase. However, during the stationary phase, the OD_600_ values of *V. parahaemolyticus* cultured alone were higher than those of *E. coli* cultured alone but comparable to those of the co-culture. This likely occurs because, during the stationary phase, the OD_600_ of *E. coli* is lower than that of *V. parahaemolyticus*. The plate counting method was further employed to analyze the actual ratio of *V. parahaemolyticus* to *E. coli* in co-cultures (Fig. 1B). At the initial time point (0 h), the CFU ratio between *E. coli* and *V. parahaemolyticus* was 110:1, which is close to the theoretical ratio of 100:1. However, by hour 14, this ratio decreased to 2:1, indicating that during co-culture conditions, the proliferation capacity of *V. parahaemolyticus* exceeds that of *E. coli*. The CS of *E. coli* had no effect on the growth of *V. parahaemolyticus*, and conversely, the CS of *V. parahaemolyticus* did not influence *E. coli* either (Fig. 1C and D). *E. coli* and *V. parahaemolyticus* were mixed in a 100:1 ratio and inoculated into 5 mL of LB broth. The mixture was then incubated at 37°C under static conditions to simulate the facultative anaerobic environment found in the intestine. The changes in CFU of both *V. parahaemolyticus* and *E. coli* in the co-culture were analyzed using plate counting methods. Over time, CFUs for both bacterial species increased; however, the proliferation rate of *V. parahaemolyticus* was faster than that of *E. coli*, resulting in a gradual decrease in the ratio between *E. coli* and *V. parahaemolyticus* (Fig. 1E and F). These results indicate that, during co-cultivation, the competitive ability of *V. parahaemolyticus* is superior to that of *E. coli*.

### Co-cultivation of *V. parahaemolyticus* and *E. coli* enhances their biofilm formation

**Fig 2:**
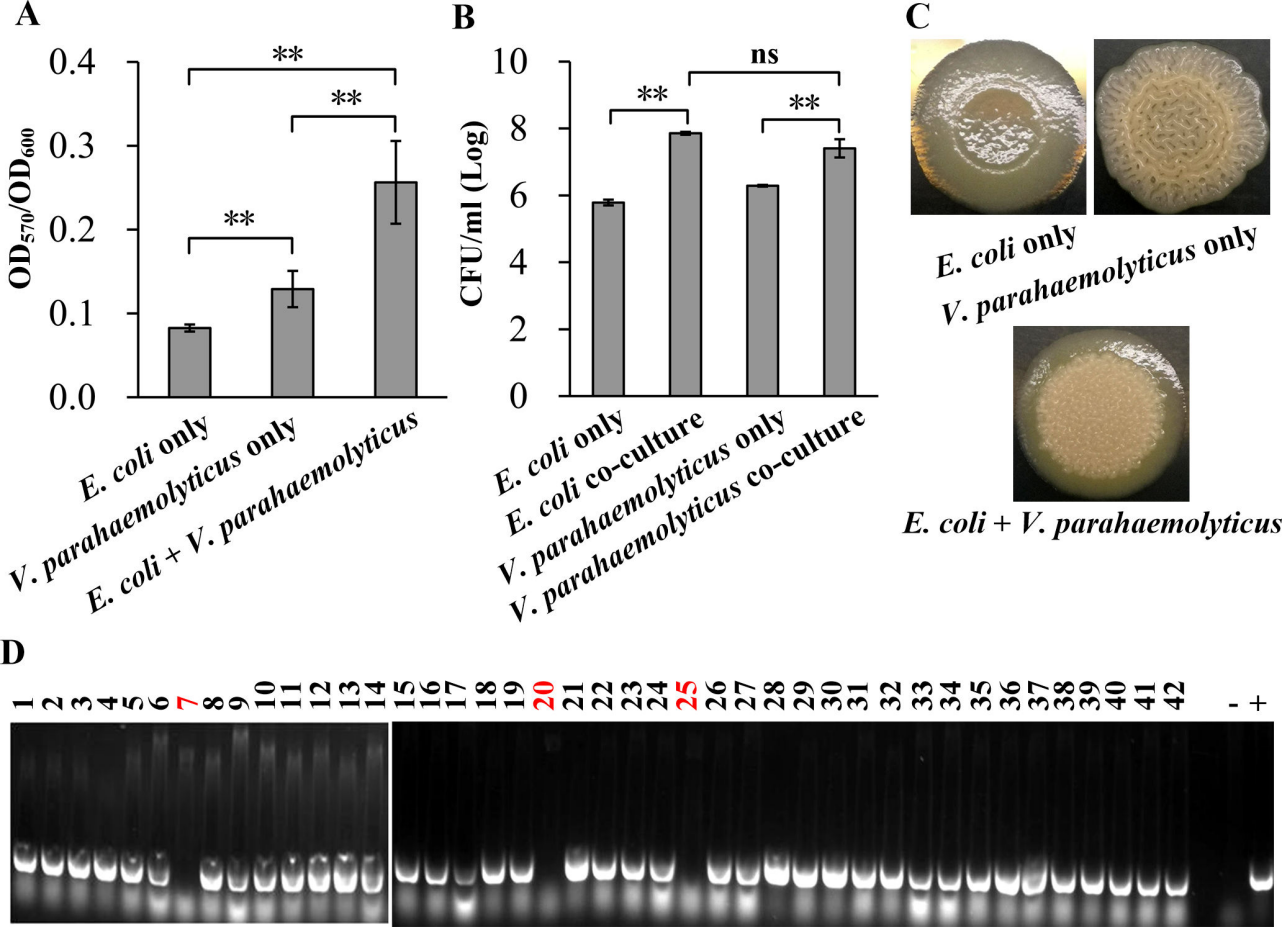
Biofilm formation of *V. parahaemolyticus* and *E. coli* under monoculture and co-culture conditions. Double asterisks suggest statistical significance (*P* < 0.01), while “ns” represents no significant difference (*P* > 0.05). (**A**) The results of CV staining for the detection of biofilm formation capabilities of *V. parahaemolyticus* and *E. coli* under monoculture and co-culture conditions. (**B**) The CFU counts of *E. coli* and *V. parahaemolyticus* in biofilms. (**C**) The colony morphology of *V. parahaemolyticus* and *E. coli* under monoculture and co-culture conditions. The pictures are representative of at least three separate experiments, with each experiment conducted in triplicate. (**D**) PCR identification of *V. parahaemolyticus* in co-culture colonies. A small amount of a mixed colony of *E. coli* and *V. parahaemolyticus* was collected from the colony morphology assay, suspended in aseptic PBS buffer, and diluted 10-fold. The diluted sample was plated onto LB agars and incubated at 37°C for 24 h. Single colonies were randomly selected for *toxR* identification using PCR. The numbers at the top of the image are identification codes for monoclonal strains, with red-marked clones indicating a negative result for the *toxR* gene. The minus sign indicates the negative control, with sterile deionized water as its template; the plus sign denotes the positive control, utilizing the genomic DNA of RIMD2210633 as its template.

The total amounts of biofilm formed by both the individual and combined cultures of *E. coli* and *V. parahaemolyticus* were quantified using CV staining (Fig. 2A). Both *E. coli* and *V. parahaemolyticus* were capable of forming biofilms; however, the biofilm formation ability of *E. coli* was significantly lower than that of *V. parahaemolyticus* (*P* < 0.01). Furthermore, the amount of biofilm formed by each species when grown individually was notably less than that observed during co-cultivation. After scraping off the biofilm, it was resuspended in sterile PBS. Subsequently, bacterial counting and classification within the biofilm were performed using the plate count method. As shown in Fig. 2B, the number of *E. coli* present in the biofilm formed under co-culture conditions was significantly higher than that found in the biofilm formed under monoculture conditions (*P* < 0.01). Similar results were also observed for *V. parahaemolyticus* (*P* < 0.01). However, no significant difference was observed between the counts of *E. coli* and *V. parahaemolyticus* within the co-cultured biofilms (*P* > 0.05). These results indicate that co-cultivation simultaneously enhances the biofilm formation capabilities of both *V. parahaemolyticus* and *E. coli*.

We further investigated the colony morphology of *E. coli* and *V. parahaemolyticus* during their individual and co-cultivation on LB agar plates. As shown in Fig. 2C, *E. coli* cultured alone formed smooth colonies, while *V. parahaemolyticus* exhibited wrinkled colonies when cultured independently. When *E. coli* and *V. parahaemolyticus* were co-cultured on LB agar plates, they produced wrinkled colonies; however, the wrinkles observed in this condition were denser compared to those formed by *V. parahaemolyticus* cultured alone. We randomly scraped a small amount of microbial colonies from the mixed culture and resuspended it in sterile PBS. After serial dilution, the suspension was plated onto LB agar, which was then incubated at 37°C until single colonies appeared. We randomly selected 42 of these colonies for colony PCR amplification targeting the *toxR* gene. As shown in Fig. 2D, only 3 clones exhibited negative amplification results, while the remaining 39 clones showed positive amplification. The proportion of *V. parahaemolyticus* exceeds 92%, indicating its predominance in the mixed colonies.

### *E. coli* CS promotes biofilm formation of *V. parahaemolyticus*

**Fig 3:**
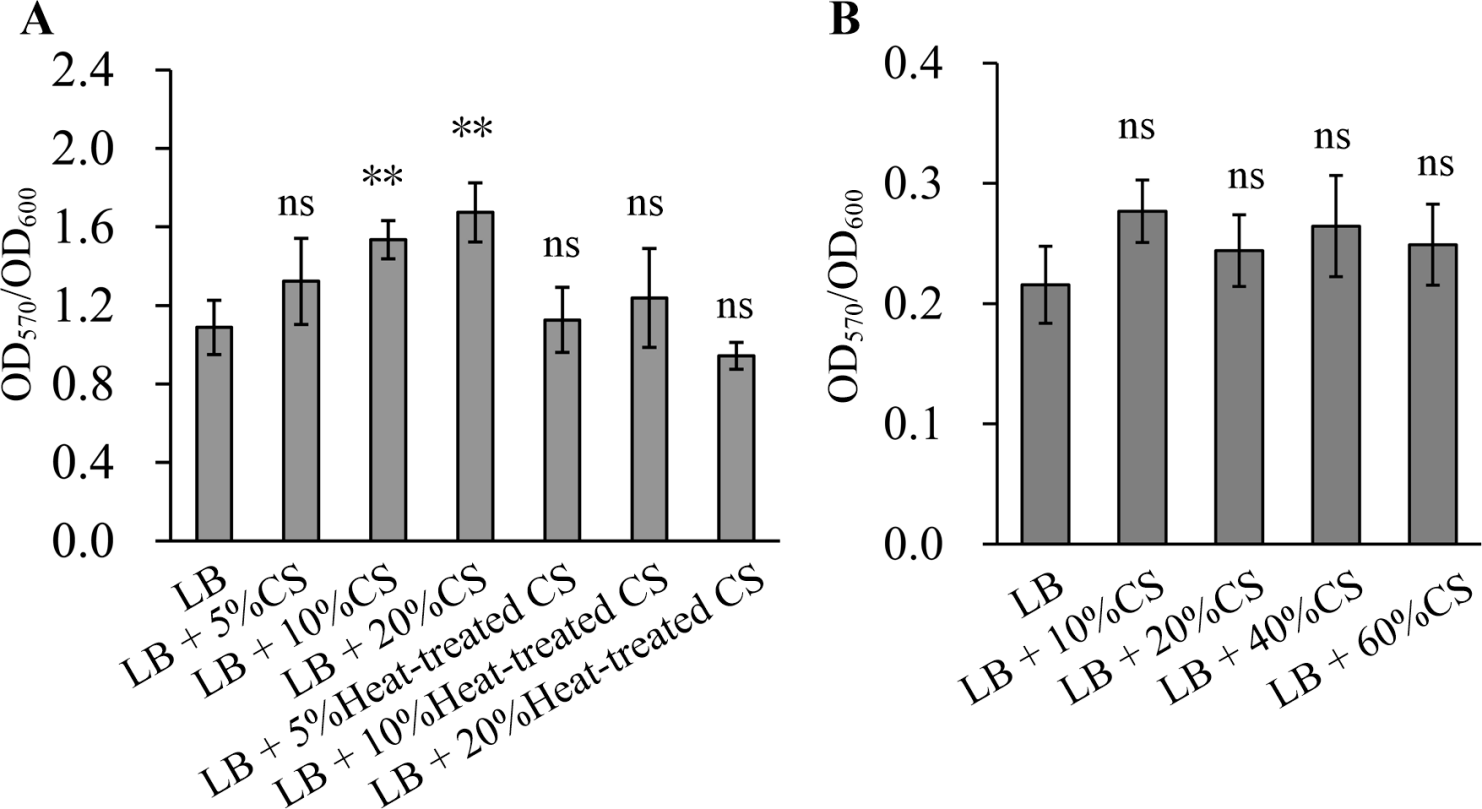
*E. coli* CS enhances biofilm formation by *V. parahaemolyticus*. Double asterisks indicate a significant difference compared to the control without added CS (*P* < 0.01), whereas “ns” represents no significant difference (*P* > 0.05). (**A**) The impact of *E. coli* CS on *V. parahaemolyticus* biofilm formation. (**B**) The impact of *V. parahaemolyticus* CS on *E. coli* biofilm formation.

The effect of *E. coli* CS on biofilm formation in *V. parahaemolyticus* was investigated using the CV staining assay. As shown in Fig. 3A, the addition of 10% and 20% *E. coli* CS to the culture medium significantly enhanced the biofilm formation ability of *V. parahaemolyticus* compared to the control without added CS (*P* < 0.01). In contrast, no significant effect was observed with 5% *E. coli* CS (*P* > 0.05). Furthermore, heat-treated CS of *E. coli* had no impact on biofilm formation by *V. parahaemolyticus* either (*P* > 0.05). Similarly, the addition of *V. parahaemolyticus* CS to the culture medium, whether present or absent, did not affect biofilm formation by *E. coli* (*P* > 0.05; Fig. 3B). These results suggest that *E. coli* CS promotes biofilm formation by *V. parahaemolyticus*.

### Effect of co-cultivation on *V. parahaemolyticus* motility

**Fig 4:**
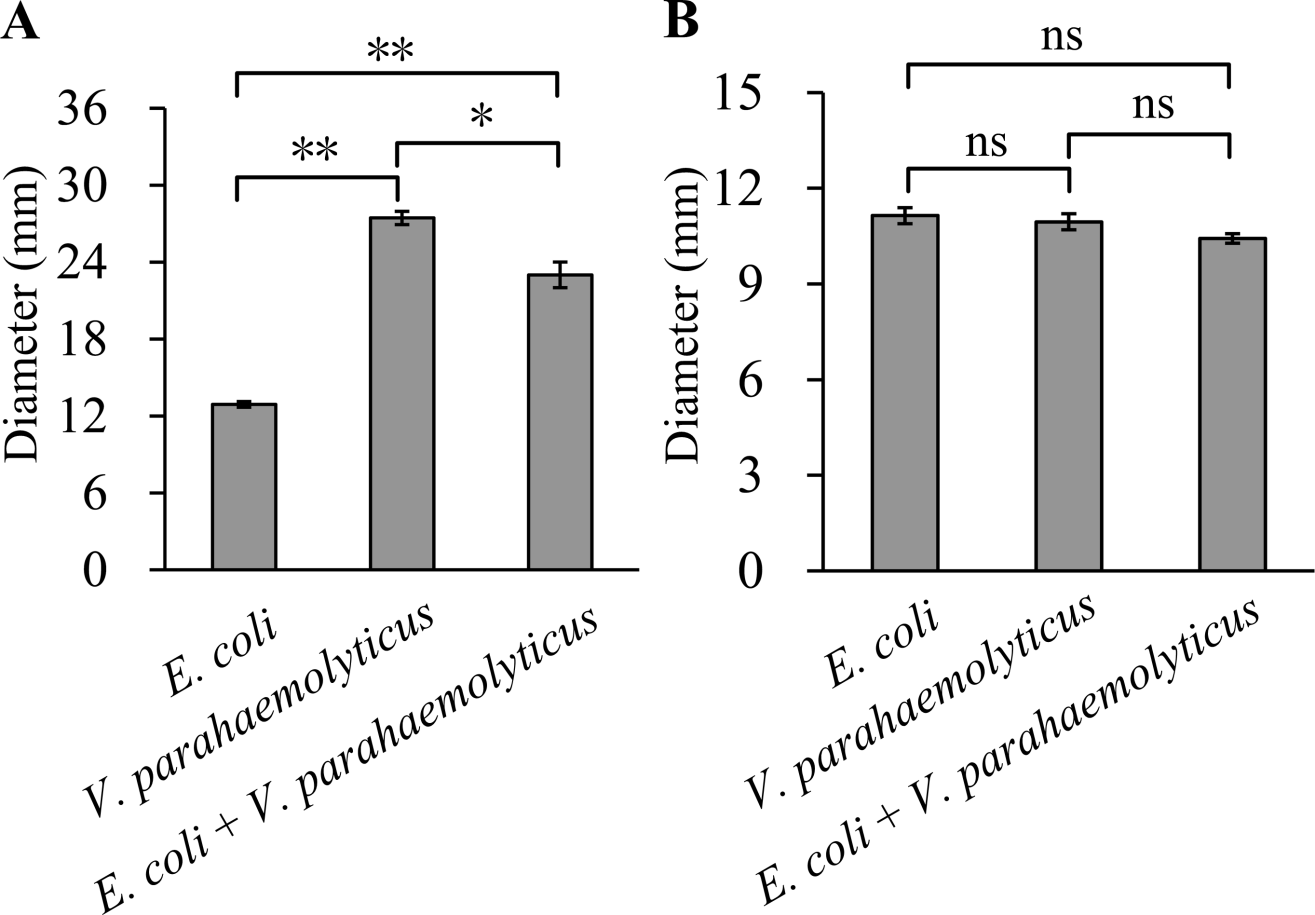
Motor capacities of *V. parahaemolyticus* and *E. coli* under monoculture and co-culture conditions. The swimming (**A**) and swarming (**B**) motility of *V. parahaemolyticus* and *E. coli* under monoculture and co-culture conditions were investigated by measuring the diameters of their respective motility zones. The data represent mean ± SD, derived from three technical replicates. Single asterisk suggests a *P* value of less than 0.05, double asterisks mean a *P* value of less than 0.01, and “ns” indicates a *P* value higher than 0.05.

The diameter of the swimming zone of *E. coli* cultured alone was significantly smaller than those of *V. parahaemolyticus* cultured alone and *E. coli* co-cultured with *V. parahaemolyticus* (*P* < 0.01; Fig. 4A). Furthermore, the swimming zone diameter of *V. parahaemolyticus* cultured alone was significantly larger than that in the co-culture with *E. coli* and *V. parahaemolyticus* (*P* < 0.05). However, there were no differences in the swarming zone diameters among *E. coli* cultured alone, *V. parahaemolyticus* cultured alone, and the co-culture of *E. coli* and *V. parahaemolyticus* (*P* > 0.05; Fig. 4B). These results suggest that co-culturing with *E. coli* reduced swimming motility of *V. parahaemolyticus*.

### Cytotoxic and adhesive activities in *V. parahaemolyticus, E. coli*, and their co-culture

**Fig 5:**
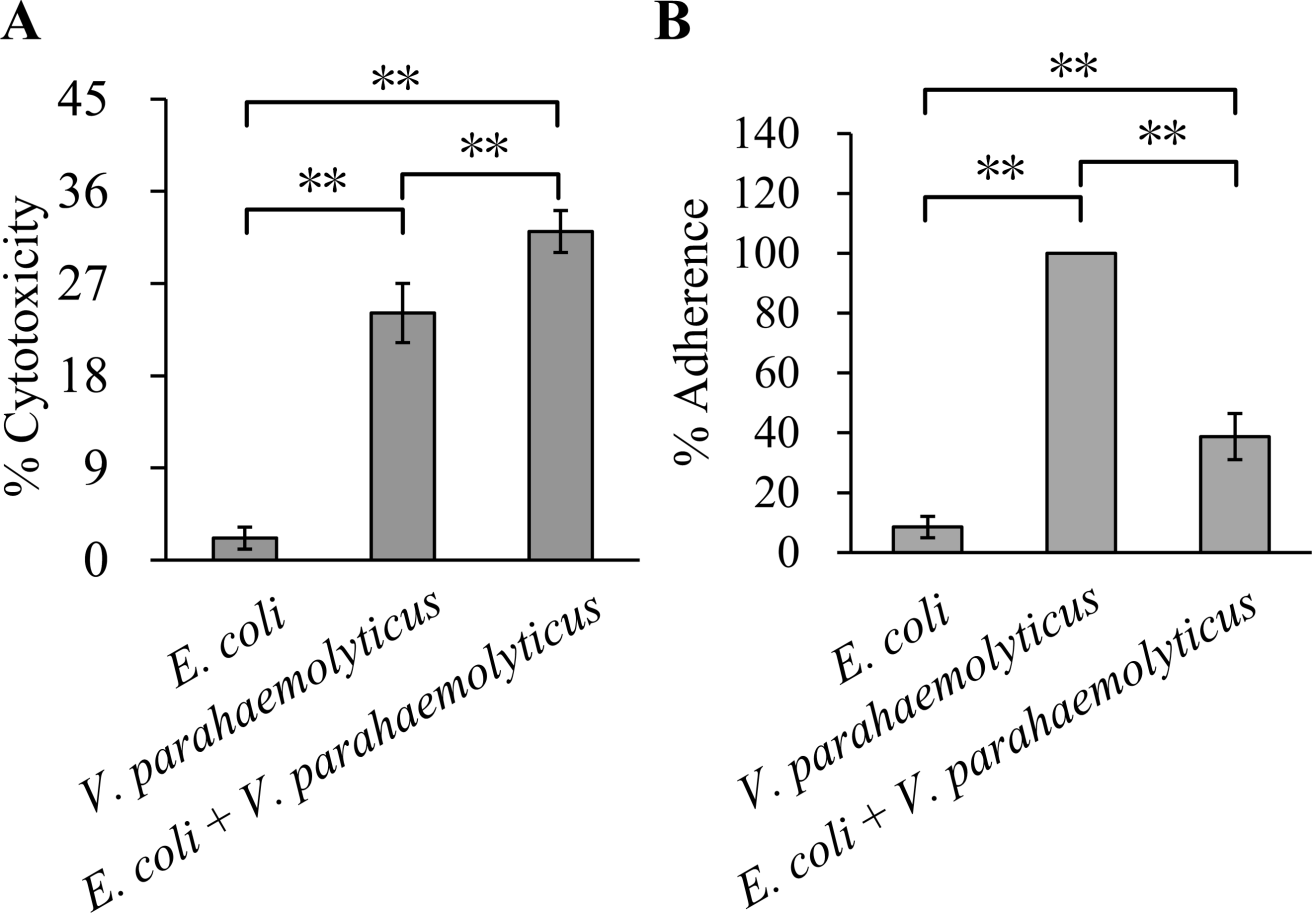
The impact of co-cultivation with *E. coli* on the activity of *V. parahaemolyticus* to HeLa cells. Values represent the mean ± SD derived from a minimum of three independent experiments, each with four replicates. Double asterisks indicate a *P* value of less than 0.01. (**A**) Cytotoxicity against HeLa cells was evaluated based on the release of LDH. (**B**) The percent adherence to HeLa cells was calculated as bacterial cells adhered/input bacterial cells and that of *V. parahaemolyticus* cultured alone was normalized to 100%.

*E. coli* cultured alone exhibited significantly lower cytotoxicity compared with *V. parahaemolyticus* cultured alone and the co-culture (*P* < 0.01; Fig. 5A). Similarly, the cytotoxicity of *V. parahaemolyticus* in monoculture was significantly lower than that of the co-culture (*P* < 0.01). However, it remains unknown whether the significantly higher cytotoxicity observed in co-culture results from an additive effect of both bacteria’s cytotoxicity or if *E. coli* enhances the cytotoxicity of *V. parahaemolyticus*. This requires further investigation. In terms of adhesion, *E. coli* cultured alone showed a significantly lower adhesion percentage than both *V. parahaemolyticus* cultured alone and the co-culture of *E. coli* and *V. parahaemolyticus* (*P* < 0.01; Fig. 5B). Moreover, the adhesion percentage of the co-culture was also significantly lower than that of *V. parahaemolyticus* cultured alone (*P* < 0.01). These results indicate that co-culturing with *E. coli* alters the adhesion of *V. parahaemolyticus* to HeLa cells.

### Co-culture with *E. coli* triggers global gene expression changes in *V. parahaemolyticus*

**Fig 6:**
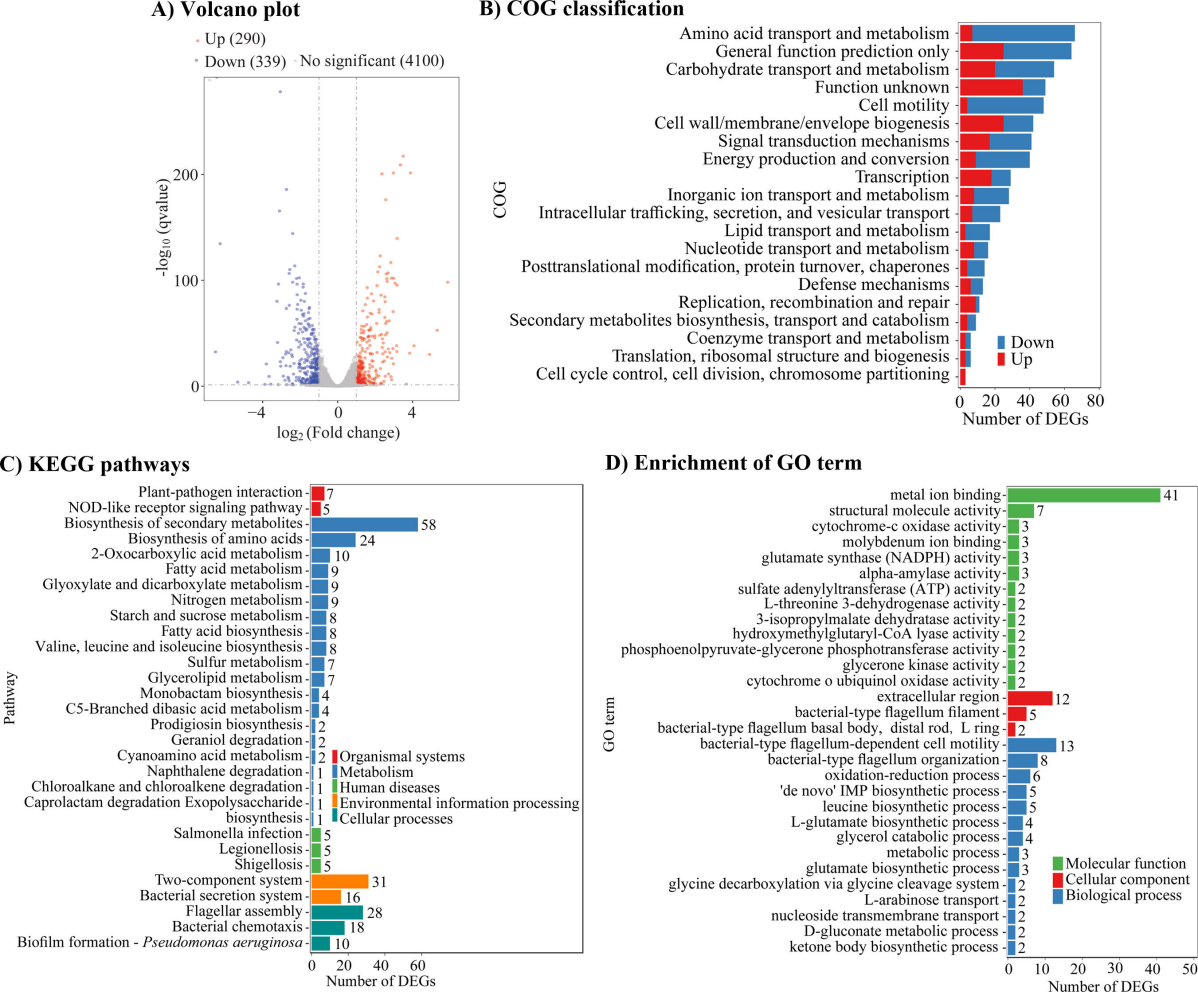
Co-cultivation with *E. coli* altered the gene expression profile of *V. parahaemolyticus*. (**A**) Volcano plot analysis. Genes significantly downregulated in response to co-cultivation with *E. coli* are marked by blue points. Orange points indicate upregulated genes, while gray points represent genes with no significant expression changes. (**B**) COG classification. The *Y*-axis shows COG categories, and the *X*-axis displays the quantity of DEGs. (**C**) KEGG pathway enrichment. The *Y*-axis represents KEGG pathways, while the *X*-axis shows the number of DEGs. The count of genes enriched in each pathway is indicated at the top of each bar. Blue bars indicate genes enriched in metabolic pathways, green bars represent human disease-related pathways, orange bars correspond to environmental information processing, red bars denote organismal systems, and dark green bars signify cellular processes. (**D**) GO enrichment analysis. The GO enrichment analysis classifies DEGs into three domains: biological processes (blue bars), cellular components (red bars), and molecular functions (green bars). The *Y*-axis indicates GO term, while the *X*-axis suggests the number of DEGs. The value atop each bar represents the count of enriched DEGs.

Gene expression in *V. parahaemolyticus* was analyzed and compared using RNA-seq between two groups: one cultured alone (the reference group) and the other co-cultured with *E. coli* (the test group). As shown in Fig. 6A, 629 DEGs were identified, comprising 290 upregulated and 339 downregulated genes. COG classification categorized the DEGs into 21 functional groups, including amino acid transport and metabolism, carbohydrate transport and metabolism, function unknown, general function prediction only, and cell motility (Fig. 6B). KEGG enrichment revealed significant associations with metabolic pathways (Fig. 6C), while GO enrichment highlighted roles in cellular components, molecular functions, and biological processes (Fig. 6D). All DEGs are listed in Table S1.

Analysis of genes of interest identified 23 polar flagellar genes and 9 lateral flagellar genes, all significantly downregulated (Table 2). Conversely, *cps* genes and two type IV pili-related genes were upregulated, whereas two *scv* genes were downregulated. Eight genes potentially related to c-di-GMP metabolism displayed significant differential expression, seven of which were upregulated. Among virulence factors, most T3SS1 genes were downregulated, while T6SS2 genes were upregulated. Only a few T3SS2 and T6SS1 genes exhibited significant expression changes. Additionally, 22 putative regulatory genes were differentially expressed.

**TABLE 2:** Selected DEGs

Gene ID	Name	Fold change	Description
Lateral flagella			
VP0769	*flgP*	0.3937	Flagellar assembly lipoprotein FlgP
VP0776	*flgC*	0.4573	Flagellar basal body rod protein FlgC
VP0777	*flgD*	0.4638	Flagellar hook assembly protein FlgD
VP0778	*flgE*	0.3902	Flagellar hook protein FlgE
VP0780		0.4278	Flagellar basal body rod protein FlgF
VP0781	*flgG*	0.1999	Flagellar basal body rod protein FlgG
VP0782	*flgH*	0.3101	Flagellar basal body L-ring protein FlgH
VP0783		0.3786	Flagellar basal body P-ring protein FlgI
VP0784	*flgJ*	0.3824	Flagellar assembly peptidoglycan hydrolase FlgJ
VP0788		0.4745	Flagellin
VP0790		0.3536	Flagellin
VP2228		0.4925	Chemotaxis response regulator protein-glutamate methylesterase
VP2229		0.4863	Chemotaxis protein CheA
VP2241	*fliN*	0.2931	Flagellar motor switch protein FliN
VP2244		0.4762	Flagellar hook-length control protein FliK
VP2245	*fliJ*	0.4865	Flagella biosynthesis chaperone FliJ
VP2254	*fliS*	0.339	Flagellar export chaperone FliS
VP2256	*fliD*	0.3596	Flagellar filament capping protein FliD
VP2257	*flaG*	0.2593	Flagellar protein FlaG
VP2258		0.454	Flagellin
VP2259		0.3478	Flagellin
VP2261		0.4323	Flagellin
VP2811		0.3388	sel1 repeat family protein
Polar flagellum			
VPA0261		0.4232	Flagellar export chaperone FlgN
VPA0266	*flgD*	0.084	Flagellar hook assembly protein FlgD
VPA0267	*flgE*	0.3687	Flagellar basal body protein FlgE
VPA0270	*flgH*	0.4564	Flagellar basal body L-ring protein FlgH
VPA1532	*fliJ*	0.1918	Flagellar export protein FliJ
VPA1534		0.4664	Flagellar assembly protein H
VPA1535		0.3483	Flagellar motor switch protein FliG
VPA1536	*fliF*	0.3883	Flagellar M-ring protein FliF
VPA1550	*fliD*	0.3546	Flagellar filament capping protein FliD
EPS			
VPA1403	*cpsA*	7.7881	Undecaprenyl-phosphate glucose phosphotransferase
VPA1404	*cpsB*	6.8748	Outer membrane beta-barrel protein
VPA1405	*cpsC*	14.4001	Polysaccharide export protein
VPA1406	*cpsD*	7.9565	Polysaccharide biosynthesis tyrosine autokinase
VPA1407	*cpsE*	8.959	Putative capsular polysaccharide synthesis family protein
VPA1408	*cpsF*	5.7932	Glycosyltransferase
VPA1409	*cpsG*	7.7471	O-antigen ligase family protein
VPA1410	*cpsH*	8.861	Putative capsular polysaccharide synthesis family protein
VPA1411	*cpsI*	7.1915	Glycosyltransferase
VPA1412	*cpsJ*	4.8642	Oligosaccharide flippase family protein
VPA1413	*cpsK*	4.207	VanZ family protein
VP1458	*scvO*	0.2427	Sugar transferase
VP1464	*scvJ*	0.3614	O-antigen ligase family protein
c-di-GMP			
VP0117	*gepA*	0.2919	EAL domain-containing protein
VP1979		3.5421	EAL domain-containing protein
VP2366		2.2753	Sensor domain-containing diguanylate cyclase
VPA0360		2.5258	GGDEF domain-containing protein
VPA0518		2.4444	GGDEF domain-containing phosphodiesterase
VPA0594		2.7021	EAL domain-containing protein
VPA0713		2.667	EAL domain-containing protein
VPA0878		2.2579	Diguanylate cyclase
Type IV pili			
VP2523		3.4947	Pilin
VP2697		2.5996	Hypothetical protein
CPS			
VP0230		2.0012	Glycosyltransferase family 4 protein
T3SS1			
VP1658	*vcrH*	0.2925	SycD/LcrH family type III secretion system chaperone VcrH
VP1662	*vcrD*	0.4933	SctV family type III secretion system export apparatus subunit VcrD
VP1667	*vopN*	0.4496	SctW family type III secretion system gatekeeper subunit VopN
VP1668	*vscN*	0.3643	SctN family type III secretion system ATPase VscN
VP1670	*vscP*	0.4003	Type III secretion system needle length determinant VscP
VP1671	*vscQ*	0.3053	SctQ family type III secretion system cytoplasmic ring protein VscQ
VP1677		0.3884	Alpha/beta hydrolase
VP1680	*vopQ*	0.4108	Type III secretion system effector VopQ
VP1682	*vecA*	0.4438	CesT family type III secretion system chaperone VecA
VP1683	*vopR*	0.3349	Type III secretion system effector VopR
VP1686	*vopS*	0.479	T3SS effector adenosine monophosphate-protein transferase VopS
VP1687		0.1873	CesT family type III secretion system chaperone
VP1690	*vscJ*	0.3794	SctJ family type III secretion inner membrane ring lipoprotein Vsc
VP1696	*vscC*	0.2736	SctC family type III secretion system outer membrane ring subunit VscC
T3SS2			
VPA1328		2.141	Hypothetical protein
VPA1370	*vopL*	0.3974	Type III secretion system effector VopL
T6SS1			
VP1410		2.7918	Hypothetical protein
VP1413	*tssK*	0.4735	Type VI secretion system baseplate subunit TssK
T6SS2			
VPA1024		5.3075	Hypothetical protein
VPA1025		5.6321	PAAR domain-containing protein
VPA1026	*vgrG*	7.8601	Type VI secretion system tip protein VgrG
VPA1027		11.277	Type VI secretion system tube protein Hcp
VPA1028	*tssH*	3.4182	Type VI secretion system ATPase TssH
VPA1029	*tssG*	6.5689	Type VI secretion system baseplate subunit TssG
VPA1030	*tssF*	5.7279	Type VI secretion system baseplate subunit TssF
VPA1031	*tssE*	7.8806	Type VI secretion system baseplate subunit TssE
VPA1032		6.5029	Protein of avirulence locus
VPA1033	*tssC*	6.7062	Type VI secretion system contractile sheath large subunit
VPA1034	*tssC*	4.4248	Type VI secretion system contractile sheath large subunit
VPA1035	*tssB*	5.2499	Type VI secretion system contractile sheath small subunit
VPA1036	*tssA*	5.7994	Type VI secretion system protein TssA
VPA1037		5.9019	Protein phosphatase 2C domain-containing protein
VPA1038	*tagF*	5.2009	Type VI secretion system-associated protein TagF
VPA1039	*tssM*	5.9351	Type VI secretion system membrane subunit TssM
VPA1040	*tssL*	6.1359	Type VI secretion system protein TssL%2C long form
VPA1041	*tssK*	5.887	Type VI secretion system baseplate subunit TssK
VPA1042	*tssJ*	8.3687	Type VI secretion system lipoprotein TssJ
VPA1043	*tagH*	6.0532	Type VI secretion system-associated FHA domain protein TagH
VPA1044		7.3203	Serine/threonine protein kinase
VPA1045		2.4163	Response regulator
VPA1046		2.4066	Hypothetical protein
Regulators			
VP0377		2.319	Helix-turn-helix transcriptional regulator
VP0692		0.4202	Transcriptional regulator GcvA
VP1004	*rsmS*	0.3954	Pleiotropic regulatory protein RsmS
VP1586		7.4169	Helix-turn-helix domain-containing protein
VP1649		2.0924	GntR family transcriptional regulator
VP2710	*scrP*	4.6114	Helix-turn-helix transcriptional regulator
VP2866		0.4536	Response regulator transcription factor
VP2885	*fis*	2.2885	DNA-binding transcriptional regulator Fis
VPA0053		0.2375	TetR/AcrR family transcriptional regulator
VPA0148		2.1744	Response regulator transcription factor
VPA0251		0.4478	LysR family transcriptional regulator
VPA0359		2.7552	Helix-turn-helix transcriptional regulator
VPA0606	*qsvR*	2.0058	AraC family transcriptional regulator
VPA0692		0.4644	LysR family transcriptional regulator
VPA0716		2.3579	Helix-turn-helix domain-containing protein
VPA0910		3.3072	Helix-turn-helix domain-containing protein
VPA0912		2.0587	LysR family transcriptional regulator
VPA0961	*acsS*	0.0129	LysR family transcriptional regulator
VPA0964	*uhpA*	2.1743	Transcriptional regulator UhpA
VPA1045		2.4163	Response regulator
VPA1446	*cpsQ*	6.2481	Helix-turn-helix transcriptional regulator
VPA1447	*cpsS*	5.7444	LuxR family transcriptional regulator

### Validation of RNA-seq data

**Fig 7:**
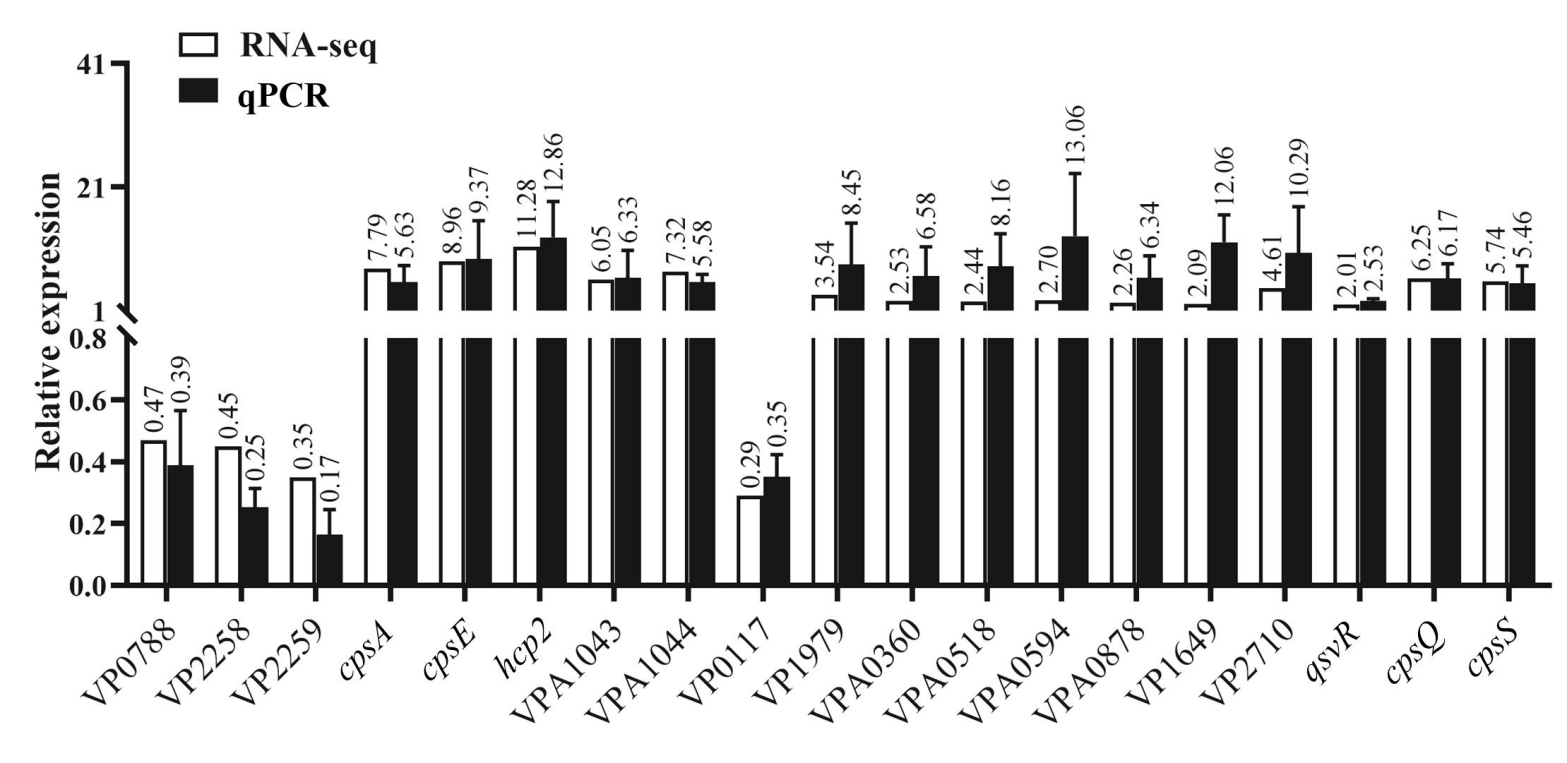
Validation of RNA-seq data via qPCR. The relative mRNA expression levels of each target gene were compared between *V. parahaemolyticu**s*** cells cultured alone and those co-cultured with *E. coli*. The 16S rRNA gene served as an internal control for normalization.

Nineteen DEGs were selected for qPCR validation (Table 2). The qPCR results correlated strongly with RNA-seq data (Fig. 7; Table 2), confirming the reliability of the transcriptomic analysis.

## DISCUSSION

This study found that *V. parahaemolyticus* exhibited stronger competitive ability than *E. coli* during co-culture. As the culture time progressed, the ratio of *V. parahaemolyticus* to *E. coli* in the co-culture medium gradually increased. Moreover, CS from either species did not inhibit the growth of the other (Fig. 1). While the genotoxin colibactin, produced by *E. coli*, has been shown to exert lethal effects on *V. cholerae* (25), we observed no bactericidal activity of *E. coli* ATCC25922 against *V. parahaemolyticus*. Similarly, another study reported that *E. coli* CS does not influence the growth of *V. vulnificus* (16), aligning with our observations. Under ideal conditions, *V. parahaemolyticus* doubles its population every 12–14 min, compared with *E. coli*, which typically doubles every 20 min. This disparity in proliferation rates likely contributes to the increasing dominance of *V. parahaemolyticus* in co-culture. Furthermore, *V. parahaemolyticus* RIMD2210633 expresses T6SS1, a system with bactericidal activity capable of eliminating competitors (26). T6SS may play a role in its competition with *E. coli*; however, only two genes within the T6SS1 cluster (VP1386-1420), comprising 35 structural genes (14), showed significant expression changes, with one being upregulated and the other downregulated (Table 2). The opposing regulation of these two genes raises questions about whether T6SS retains functional activity under the tested conditions. Notably, T6SS1 is most active in warm marine-like environments (26), suggesting its role in *V. parahaemolyticus-E. coli* competition under our experimental conditions requires further investigation.

CV staining results indicated that co-cultivation simultaneously enhanced biofilm formation in both *V. parahaemolyticus* and *E. coli* (Fig. 2A). Furthermore, CS from *E. coli* promoted biofilm formation by *V. parahaemolyticus*, while CS from *V. parahaemolyticus* had no effect on *E. coli* biofilm formation (Fig. 3). These findings suggest that during infection, *V. parahaemolyticus* may detect specific molecules secreted by *E. coli* in the intestinal environment, triggering enhanced biofilm formation and increased resistance to environmental stressors. In contrast, *E. coli* may protect itself via biofilm formation when challenged by competitors. Previous studies reported that co-culturing with *E. coli* reduced biofilm formation in *V. vulnificus* but enhanced it in *V. cholerae* (16, 27), highlighting significant variability in interaction mechanisms and outcomes among intestinal pathogens and gut microbiota. Furthermore, co-cultivation altered bacterial colony morphology on solid surfaces, with *V. parahaemolyticus* dominating in the co-cultured colonies (Fig. 2C and D). This observation suggests that *V. parahaemolyticus* likely eliminates a substantial proportion of *E. coli* within the colonies via its T6SS1, which exhibits heightened activity under plate culture conditions (26).

*V. parahaemolyticus* possesses polar and lateral flagella, which mediate swimming motility in liquids and swarming motility on surfaces, respectively (28). Loss of polar flagellum genes results in impaired swimming motility and reduced biofilm formation in *V. parahaemolyticus* (29). Notably, flagella-mediated motility and biofilm formation in *V. parahaemolyticus* typically exhibit an inverse relationship: factors promoting biofilm formation often inhibit motility (30). This study found that co-cultivation with *E. coli* reduced swimming motility of *V. parahaemolyticus* (Fig. 4). This contrasts with the observed enhancement in biofilm formation, aligning with expected inverse relationship. These findings suggest that flagella-mediated motility plays a key role in the interaction between *V. parahaemolyticus* and *E. coli*, a conclusion supported by an analogous study in *V. cholerae* and *E. coli* (27). Furthermore, co-culturing with *E. coli* might enhance *V. parahaemolyticus* cytotoxicity but reduce its adhesion to HeLa cells (Fig. 5), suggesting that interspecies interactions may modulate the pathogenicity of *V. parahaemolyticus*.

We further employed RNA-seq to investigate the molecular mechanisms underlying the phenotypic changes in *V. parahaemolyticus* during interaction with *E. coli*. The results revealed differential expression of 629 genes in *V. parahaemolyticus* (290 upregulated, 339 downregulated), affecting diverse cellular pathways (Fig. 6). Notably, the *cpsA-K* cluster was significantly upregulated, whereas *scvO* and *scvJ* were downregulated (Table 2). The *cpsA-K* and *scvA-O* clusters, responsible for EPS production in *V. parahaemolyticus* biofilms, are positively associated with biofilm formation (31). However, only *cpsA-K* influences colony morphology on agar plates (31). c-di-GMP post-transcriptionally regulates motility, biofilm formation, and pathogenesis (32). It is synthesized by guanylate cyclases that contain a GGDEF domain and degraded by phosphodiesterases that possess either an EAL or HD-GYP domain (32). Among eight DEGs encoding these enzymes, seven were upregulated, while *gepA* was downregulated (Table 2). GepA is a phosphodiesterase that degrades c-di-GMP, suppresses biofilm formation, and enhances swimming motility in *V. parahaemolyticus* (33, 34). The functions of the remaining seven genes remain uncharacterized. Two type IV pili genes (VP2523 and VP2697) and one capsular polysaccharide (CPS) gene (VP0230) were upregulated in co-cultured *V. parahaemolyticus* cells (Table 2). Type IV pili promote biofilm formation, while CPS inhibits this process (35, 36). Transcriptomic data also revealed significant downregulation of 23 polar and 9 lateral flagellar genes (Table 2), indicating that co-cultivation modulates *V. parahaemolyticus* motility and biofilm formation via flagellar synthesis. Collectively, these findings indicate that *E. coli-V. parahaemolyticus* interactions alter biofilm formation and motility in *V. parahaemolyticus* through transcriptional reprogramming of genes related to EPS production, c-di-GMP synthesis, type IV pili, and flagellar function.

A total of 14 genes related to T3SS1 were downregulated, while 23 genes associated with T6SS2 were upregulated (Table 2). T3SS1 is primarily linked to cytotoxicity in cell lines such as HeLa, Caco-2, and RAW264.7 cells, and it induces apoptosis and lethality in mice (37). Paradoxically, despite the downregulation of T3SS1-related genes, cytotoxicity was significantly enhanced after co-culture with *E. coli* (Fig. 5A), suggesting unresolved molecular mechanisms requiring further investigation. In contrast, T6SS2 is implicated in cell adhesion and biofilm formation (26, 38), implying that altered adhesion activity in *V. parahaemolyticus* post-co-culture may partly stem from T6SS2 upregulation. Furthermore, two T3SS2 genes exhibited divergent expression changes, with one upregulated and the other downregulated (Table 2). The T3SS2 genetic cluster (VPA1320-1370) contains 51 structural genes (14), and the opposing regulation of only two genes within this cluster raises questions about its functional impact. Does this partial regulation compromise T3SS2 activity? Further studies are needed to resolve this ambiguity.

RNA-seq data revealed significant expression changes in 22 genes encoding putative regulatory proteins during *E. coli* and *V. parahaemolyticus* interactions (Table 2). These include global regulators such as LysR family transcriptional regulators (VPA0251, VPA0692, VPA0912, and AcsS), a LuxR family transcriptional regulator (*cpsS*), an AraC family transcriptional regulator (QsvR), a GntR family transcriptional regulator (VP1649), and a TetR/AcrR family transcriptional regulator (VPA0053). CpsQ, CpsS, and ScrP are c-di-GMP binding proteins; however, CpsQ and CpsS require c-di-GMP for regulatory activity, whereas ScrP functions independently of c-di-GMP (39). CpsQ and CpsS promote biofilm formation, while ScrP has little effect on this process (39). CpsQ additionally regulates T6SS2 genes (40). QsvR works with OpaR and AphA to regulate diverse cellular pathways, including biofilm formation, virulence factor expression, and motility (41–45). AcsS promotes swimming and swarming motility while inhibiting virulence genes linked to T6SS2 and TDH (46–48). The roles of other regulators remain uncharacterized, warranting further investigation.

In summary, this study demonstrated that *V. parahaemolyticus* outcompetes *E. coli* in co-culture due to superior proliferation efficiency. The CS from neither species inhibited the other’s growth. Co-cultivation enhanced biofilm formation in both species, with *E. coli* CS promoting biofilm formation by *V. parahaemolyticus*. However, *V. parahaemolyticus* dominated mixed colonies. Co-culture reduced swimming motility and adhesion activity of *V. parahaemolyticus* while enhancing its cytotoxicity against HeLa cells. RNA-seq analysis revealed 629 DEGs in co-cultured *V. parahaemolyticu*s (290 upregulated, 339 downregulated). Downregulated genes included those associated with flagella and T3SS1, while upregulated genes linked to EPS, type IV pili, and T6SS2. Additionally, 8 c-di-GMP metabolism-related genes were differentially expressed (seven upregulated), and 22 putative regulators showed altered expression, indicating tight transcriptional control during interspecies interaction. The primary limitation of this study is that it only investigated the interactions between a single strain of *E. coli* and *V. parahaemolyticus* under *in vitro* conditions, focusing on the effects of these interactions on the functional phenotypes and gene expression in *V. parahaemolyticus*. Future research could explore how *E. coli* interacts with *V. parahaemolyticus* within the intestinal environment. Additionally, we employed *E. coli* as a representative intestinal bacterium to study its effect on *V. parahaemolyticus*. However, using more natural, physiologically relevant gut bacteria should be prioritized in future work to obtain more applicable insights. Nevertheless, this study may serve as a foundation for subsequent research exploring the interactions between commensal bacteria in the intestinal tract and human gut pathogens.

## Data Availability

The original data are available in the article and its supplementary materials. The raw data of RNA-seq are deposited in the NCBI repository under accession number PRJNA1282890.

## SUPPLEMENTAL MATERIAL

Table S1 spectrum.00789-25-s0001.xls All DEGs.

ASM does not own the copyrights to Supplemental Material that may be linked to, or accessed through, an article. The authors have granted ASM a non-exclusive, world-wide license to publish the Supplemental Material files. Please contact the corresponding author directly for reuse.
